# The Influence of Colorants, Flavorants and Product Identity on Perceptions of Naturalness †

**DOI:** 10.3390/foods8080317

**Published:** 2019-08-04

**Authors:** Tyler Murley, Edgar Chambers

**Affiliations:** Center for Sensory Analysis and Consumer Behavior, Kansas State University, 1310 Research Park Dr., Manhattan, KS 66502, USA

**Keywords:** Food naturalness, perception, natural, artificial, food additives

## Abstract

Natural foods are important to consumers, yet frustrating to producers due to the lack of a formal definition of “natural”. Previous work has studied how consumers define naturalness and how they rate the naturalness of various products, but there is a gap in knowledge relating to how color and flavor additives impact perceptions. The objective of this study was to understand how colorants and flavorants on ingredient statements affect perceptions of naturalness. An online survey was launched in the United States, United Kingdom, and Australia to determine how consumers perceive products with ingredient statements containing different combinations of artificial and natural colors and flavors when shown with and without the product identity. Results showed that consumers look at the whole product primarily to make decisions about naturalness, but also consider other factors. Products derived from plants and products with natural colors and flavors were perceived to be the most natural. Artificial flavors may be more acceptable than artificial colors due to negative health perceptions and labeling rules associated with colors. Additionally, factors like ingredient familiarity and processing likely influence consumers when making decisions about product naturalness. Males, Millennials, and educated participants have higher naturalness scores than other participants in their respective demographics.

## 1. Introduction

In the US and in many other countries, packaging requires that ingredients are listed on the package in descending order by weight with the ingredients listed at the end comprising the smallest percentage of the total formula [1]. For most ingredients, the common name is listed on the statement. Colors can be listed by their specific names, like FD&C Yellow No. 5 or just Yellow 5, if they are certified colors or listed by their common names, like Vegetable Juice for Color or natural color, if they are non-certified colors. Flavors are listed as “artificial flavor” and/or “natural flavor” on ingredient statements [2]. In some countries, the e-number is used instead of the actual name. Previous research has been conducted on consumer perceptions of food labels and ingredients. This work includes the use of and beliefs about nutrition labels, effect of product name and descriptions on perception, influence of nutrition labeling on expectations and sensory perceptions, impact of label and ingredient claims on expectations of liking, effect of pictures and photographs on expectations and perceptions, and impact of organic certification logos on consumers’ willingness to pay and preference for food products [3,4,5,6,7]. Most of this work focuses on label claims and nutrition labels leaving the ingredient statement relatively unstudied. Recent work [7] has shown that consumers have a clear mistrust of ingredients they do not know or recognize (e.g., sorghum flour) or ingredients that are described by chemical names and numbers instead of common names (e.g., sodium bicarbonate instead of baking soda).

Color and flavor additives are important and controversial ingredients used in many processed food and beverage products in the United States. According to the FDA, artificial flavor is “any substance, the function of which is to impart flavor, which is not derived from a spice, fruit or fruit juice, vegetable or vegetable juice, edible yeast, herb, bark, bud, root, leaf or similar plant material, meat, fish, poultry, eggs, dairy products, or fermentation thereof” [1]. They state that natural flavors come from essential oils, oleoresins, essences/extractives, protein hydrolysates, distillates, or products of roasting, heating, or enzymolysis containing flavor derived from the sources listed above, in which artificial flavors cannot be derived [1]. Colors, on the other hand, are only defined as color additives, which are dyes, pigments, or other substances that impart color [8].

There has been a large amount of work conducted on how food color influences perceptions of food and beverages. In a study from 1980, Dubose et al. added congruent and incongruent colors to fruit flavored beverages [9]. They found that participants more frequently misidentified the flavor of the beverage when the color was incongruent with the flavor of the beverage. They also found that color intensity affected the flavor acceptance of colored beverages and colored cakes. Similarly, Zampini found that people correctly identified the flavor of aqueous solutions more often when the color corresponded with their expectations [10]. The lime solution was correctly identified more frequently when the solution was green or colorless, for example. Correct identification did not occur with strawberry, however. This occurs because colors tend to be associated with specific flavors. Orange color was associated with orange flavor, yellow with lemon flavor, and green with lime flavor. Associations for the color red, however, were more complex and participants related red to strawberry, raspberry, and cherry flavor. Food color can also influence expectations prior to tasting. Zellner and Durlach found that brown colored lemon and mint beverages were expected to be less refreshing and clear beverages were expected to be more refreshing [11]. Brown-colored lemon and mint beverages were found to be less refreshing than other colors after tasting as well. The color of the beverage affected expectations of flavor intensity, though there were fewer significant differences after tasting. They also found that color affects expectations of liking and actual liking. Spence published a comprehensive review of color perception studies discussing the influence of color on basic taste and flavor perception, the influence of color on aroma perception, the influence of color on detection thresholds, the influence of color on flavor identification, and the influence of color on expectations [12].

Research has also been conducted to study the relationship between artificial colors and health. When comparing artificial colors and sweeteners, participants perceived significantly more risks with colors [13]. Wąsowicz found that Polish consumers believe that unhealthy products contain artificial colors along with being high in fat and calories [14].

Compared to color additives, little work has been done to study how flavor additives affect perceptions. According to Nielsen, 62 and 61% of global respondents avoid artificial flavors and artificial colors, respectively [15]. They also report that a lack of artificial colors and flavors and the presence of natural flavors is important to global consumers when making purchasing decisions [16]. Additionally, FONA reports that 69% of American consumers believe that products without artificial colors and flavors are more important than “natural” products [17]. It is clear that color and flavor additives are important to consumers.

Various authors have shown that natural concerns are important in food choices [18,19], but the interpretation of naturalness is inconsistent. It is confused with organic or locally grown [20] and often is assumed to be more healthful by consumers [21,22,23]. However, those beliefs are not consistent from country to country where various authors have found that natural either is not as relevant or there is less association with health [24,25]. Research also has shown that labeling foods with a Protected Designation of Origin (PDO), a Euorpean Union designation for specific production areas of certain foods, does not necessarily improve liking scores [26] Other motivations for desiring more natural products include moral and ethical concerns [21,27,28].

Although there has been plenty of research studying how color affects perceptions, there is a gap in knowledge related to how color and flavor additives affect perceptions of product naturalness. Since there is no formal definition of natural, it is necessary for academics and product developers to get a better understanding of this vague term and how products with artificial and/or natural colors and flavors fit into the consumer definition. This research was conducted to address this gap in knowledge. Without such knowledge, scientists, manufacturers and government cannot begin to explain to consumers how various ingredients and products fit within the scope of natural or not. The objectives of this study were (1) to understand how products with artificial and/or natural colors and flavor additives affect consumer perceptions of naturalness and (2) to understand what consumers believe are appropriate sources of natural color and flavor additives.

## 2. Materials and Methods

A standardized online questionnaire was used by consumers to gather data for this research in three countries: United States of America (USA), the United Kingdom (UK), and Australia (AUS).

### 2.1. Questionnaire

A questionnaire was developed based on questions asked in similar surveys. The questionnaire was reviewed by scientists in sensory sciences and consumer behavior and changes were made. In addition, the survey was used by 10 consumers to better understand readability, understanding and flow. Once minor changes were made, an initial pilot study was conducted to assure that that no further changes were needed and to gauge completion time.

For the final study, an online survey was launched using Qualtrics Survey Software (Provo, UT, USA) and participants were compensated using a reward system offered by Qualtrics. Participants were shown eight statements from four food products, which each being shown twice. The first time the statement was shown, participants were only shown the ingredient statement with no other information and were asked to rate how natural they perceived the food to be. This was rated on a 9-point scale anchored with “1—Not at All Natural” and “9—Extremely Natural”. After seeing all four statements, they were shown the same statements, this time being informed of the identity of the product. The four statement were chosen because they have different combinations of artificial and natural colors and flavors. Product ingredient statements included Strawberry Puree (Natural Color, Natural Flavor), Flamin’ Hot Cheetos^®^ (Artificial Color, Natural Flavor), Gummy Candy (Artificial Color, Artificial Flavor), and Blueberry Yogurt (Natural Color, Artificial Flavor). In addition to these statements, participants were shown two check all that apply (CATA) lists and were asked to select all the sources they believe that natural colors and natural flavors for food can come from.

Respondents were also asked various demographic questions including gender, age, race/ethnicity (using race demographics commonly used in each country/region), education level, income (using income brackets commonly used in each country/region), and number of children. They were also asked how often they read ingredient statements, how often they pay attention to the source of coloring/flavors on labels, importance of color/flavor on the label, and likelihood to purchase based on the presence of artificial colors/artificial flavors. Participants were also asked how often they consume various foods from a list of foods that contains color additives, in CATA format. Items from the Health and Taste Attitudes Scale were included to further segment participants.

### 2.2. Consumers

One thousand participants from the United States, the United Kingdom, and Australia were recruited. Predominantly English-speaking countries were chosen so that comparisons could be made within a common language, with slight variations in spelling and wording. Gender, age, and estimate of household grocery shopping were used to recruit participants. Three quotas categories (gender, age, and estimate of household grocery shopping) were employed to recruit potential participants: gender (50% males, 50% females), age (20–25% for 18–23, 24–41, 42–52, and 53–73; 10% or less for 74 years or older), and estimate of household grocery shopping. Participants were not included if they were under 18 years of age or if they did fewer than 40% of the grocery shopping for their household. Age generations were used instead of traditional age brackets to form more accurate conclusions about perceptional differences by age group. The generational groups, from youngest to oldest, were Centennials/Gen Z, Millennials, Gen X, Baby Boomers, and the Silent Generation. The final demographic characteristics of the sample in each country are given in Table 1 and Table 2.

### 2.3. Analysis

Excel (Microsoft Office Pro ver. 2013) was used to calculate means and percentages, for descriptive statistics, and for chi-square tests for significance (p-vales less than 5% were considered significant). XLSTAT (Addinsoft, New York, NY, USA) was used for Analysis of Variance and Correspondence Analysis for CATA data. Prior to analysis, 9-point scales were converted to 3-point scales to understand existing trends in perceptions. The scale was reduced to 1—Unnatural, Neither natural nor unnatural, and 3—Natural. Respondents with incomplete surveys were excluded before analysis. Some respondents are inattentive and may answer questions without thinking or simply check boxes without reading the question [29,30]. Because of this, a fake or “cheater” question (e.g., [31]) was included in the survey. Participants who reported consuming ‘Live worms’ or ‘Pickled chicken’ in the past week were excluded from the analysis as these would be extremely unusual foods for the three countries tested. After exclusion, 969 respondents from Australia, 959 respondents from the UK, and 932 respondents from the US were included in the analysis. Analyses for each of the demographic subsets was done and is discussed, but specific data are not shown.

## 3. Results

### 3.1. United States

**Figure 1:**
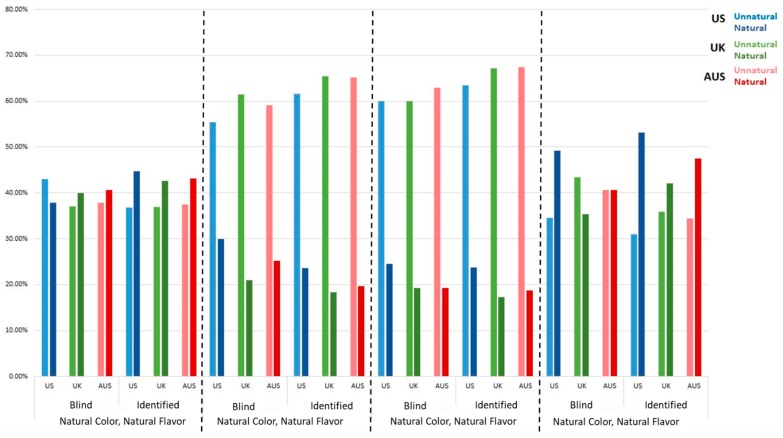
Percentage of US, UK, and AUS respondents who rated products represented by ingredient statements with natural or artificial colors or flavors as natural or unnatural.

Both of the fruit-based products were perceived as the most natural and were considered more natural when the product identity was revealed. Fifty-three percent of US respondents rated the Blueberry yogurt (Natural Color, Artificial Flavor) as natural (Figure 1). This result was not significantly different from the unidentified version of this statement, which was perceived as natural by 49% of respondents. There was also no significant difference between the unidentified Blueberry Yogurt and the identified Strawberry Puree (Natural Color, Natural Flavor), which was perceived as natural by 45% of respondents. Participants perceived the Puree to be less natural when unidentified and the percentage of natural ratings dropped to 38%. There was no significant difference between the unidentified Flamin’ Hot Cheetos^®^ (Artificial Color, Natural Flavor) and the unidentified Gummy Candy (Artificial Color, Artificial Flavor), which were perceived as natural by 30 and 25% of respondents, respectively. The unidentified Gummy Candy also was not rated significantly different from the identified Flamin’ Hot Cheetos^®^ and the identified Gummy Candy, which were both perceived as natural by 24% of American respondents.

All statements, whether identified or unidentified, were perceived to be more natural by males and less natural by females. Millennials also perceived all of the statements to be more natural than any other generational groups. These differences were significant for every statement but the identified Blueberry Yogurt. Centennials, Silent Generation, and Baby Boomers generally rated all statements as less natural. Respondents with college or post-graduate degrees perceived the statements to be more natural. These differences, however, were only significant for the unidentified Strawberry Puree, unidentified Gummy Candy, identified Cheetos, and identified Gummy Candy. Respondents with higher incomes also rated the statements as more natural, though these differences were not significant for the unidentified and identified Blueberry Yogurt statements. Parents perceived products as more natural than participants with no children. There was no significant difference, however, for the identified Blueberry Yogurt statement. Race was not a good predictor of naturalness perceptions and there were no significant differences for any statement.

American respondents who read ingredient statements more often when making purchases perceived the products to be more natural. Additionally, respondents who reported that they pay attention to the source of color and the source of flavor more often when making purchases perceived the products to be more natural. There were, however, no significant differences for the identified Yogurt for these three questions. Respondents who stated that the source of color and the source of flavor in foods and beverages was important perceived the products to be more natural. There was no significant difference for the unidentified Yogurt for flavor (though it contains artificial flavors) and there were no significant differences for the identified yogurt for color and flavor. Thirty-eight percent of respondents were likely not to purchase products with artificial colors and 37% were likely not to purchase products with artificial colors. Despite this, these respondents perceived all of the products to be more natural that those whose purchase decisions are not affected by artificial colors and flavors.

Participants were also asked how frequently they consumed commonly colored and flavored foods. These foods include Toaster Pastries, Hard Candy, Cookies, Ice Cream, Popsicles, Flavored Gelatin, Chewing Gum, Breath Mints, Breakfast Cereal, Flavored Crackers, Salad Dressing, Fruit Yogurt, Fruit Juice, Soda, Energy Drinks, and Sports Drinks. Generally, participants who frequently consumed these products perceived the products as more natural. There were significant differences in consumption for all of these foods/beverages for the unidentified Puree and the identified and unidentified Gummy Candy. There were no significant differences in consumption for Salad Dressing and Fruit Juice for the unidentified Cheetos; Ice Cream, Cereal, Dressing, and Soda for the unidentified Yogurt; Dressing for the identified Puree; Dressing and Juice for the identified Cheetos; and Ice Cream, Gum, Cereal, Dressing, Juice, and Soda for the identified Yogurt. Frequency of Salad Dressing consumption was a poor predictor of naturalness ratings. Surprisingly, some respondents who agree that they are very particular about the healthiness of food perceived the products as more natural perhaps because they read labels more often and are more familiar with various ingredients. There were no significant differences for the unidentified Cheetos, identified Cheetos, and identified Yogurt statements. The former two were believed to be unnatural by most of the participants and the latter was perceived to be natural by most of the participants. Twenty-nine percent of respondents agree that artificially colored and artificially flavored foods are not harmful for health and they perceived all products as more natural. Those that believe that artificial colors and flavors are harmful for health perceived products to be less natural. Participants that stated that they try to eat foods that do not contain additives perceived products to be more natural. There were no significant differences, however, for the identified and unidentified Yogurt. Respondents who stated that they would like to eat only organically grown vegetables, that they look for only Non-GMO (Genetically Modified Organisms) ingredients in the foods they eat, and that they always look for natural ingredients in the snack foods that they eat perceived all statements to be more natural. There was no significant difference for the identified Yogurt for participants who look for natural ingredients in snack foods.

### 3.2. United Kingdom

The identified Strawberry Puree (Natural Color, Natural Flavor) and the identified Blueberry Yogurt (Natural Color, Artificial Flavor) were perceived to be natural by the largest percentage of UK respondents (Figure 1). The former was rated natural by 43% of respondents and the latter by 42% of respondents (Figure 1). There was no significant difference between these two and the unidentified Strawberry Puree, which was perceived as natural by 40% of respondents. The unidentified puree was also not significantly different from the unidentified Blueberry Yogurt statement (35% natural). There were no significant differences in naturalness perceptions for the unidentified Flamin’ Hot Cheetos^®^ (Artificial Color, Natural Flavor), unidentified Gummy Candy (Artificial Color, Artificial Flavor), and the identified Cheetos, which were considered natural by 21, 19, and 18% of participants, respectively. There was also no significant difference between the identified Cheetos and the identified Gummy Candy (17% natural).

In general, males gave higher naturalness ratings than females. These differences were not significant, however, for the unidentified and identified Puree and the unidentified Yogurt. Millennials perceived the products to be more natural, but there were no significant differences for the identified Puree and identified Yogurt. Respondents with more education and more income perceived the products to be more natural than other groups in these demographics. There were no significant differences for the unidentified Puree for income and for the identified and unidentified Yogurt and the identified Puree for both demographics. Generally, parents perceived products to be more natural than participants without kids, but there were no significant differences for the identified and unidentified Puree and the identified and unidentified Yogurt. These four statements had the least amount of significant differences for all of these demographics. Race was not a good predictor of perceptions of naturalness.

UK respondents who read ingredient statements more frequently when making purchases perceived all products to be more natural. Similarly, respondents who pay attention to the source of color and flavor more often were more likely to perceive the products as more natural. There was no significant difference, however, for the identified and unidentified Yogurt and the identified Puree for attention to source of color. Both of these products contain natural colors. Respondents who reported that the source of color was important to them had higher perceptions of naturalness, though there were no significant differences for the identified Cheetos (which contain artificial colors) and the identified and unidentified Yogurt. For flavor source, the identified gummy candy (which contains artificial flavors) was the only product with significant differences and perceptions of naturalness were higher for respondents who report flavor source as important. Thirty-three percent and 44% of UK respondents stated that they were likely not to purchase products with artificial colors or artificial flavors, respectively. Those who were more likely not to purchase artificially colored or flavored products, however, perceived most products as more natural. There were no significant differences for the unidentified Puree for color and for the identified and unidentified Yogurt for color and flavor.

None of the products had significant differences in naturalness perceptions based on consumption of the commonly colored foods used in the survey. Generally, UK respondents who frequently ate commonly colored/flavored foods perceived the statements to be more natural. The identified Yogurt had the fewest significant differences. There was no significant difference for consumption of Cereal and Soda for the unidentified Puree; Cookies and Cereal for the unidentified Cheetos; Cereal, Fruit Juice, and Soda for the unidentified Gummy Candy; Cookies, Cereal, and Soda for the unidentified Yogurt; Cereal for the identified Puree; Cookies, Cereal, Fruit Juice, and Soda for the identified Cheetos; Cereal and Fruit Juice for the identified Gummy Candy; and Hard Candy, Gum, Breath Mints, Cereal, Fruit Juice, Soda, Energy Drinks, and Sports Drinks for the identified Yogurt. Breakfast Cereal, Soda, and Fruit Juice were poor predictors of naturalness perceptions for UK respondents. Those who agree that they are very particular about the healthiness of food perceived the unidentified and identified Puree and the identified Cheetos to be more natural. Twenty-three percent of UK respondents believe that artificially colored and artificially flavored foods are not harmful for health. These people perceived all of the products to be more natural than those who believe that artificially colored and flavored foods are harmful for health. Participants who state that they try to eat foods that do not contain additives perceived the products to be more natural, but there were no significant differences for the unidentified and identified Puree, and the identified Yogurt. Participants who would like to eat only organically grown vegetables perceived all of the products to be more natural. Those who look for only Non-GMO ingredients also perceived the products to be more natural. There were no significant differences for the unidentified and identified Puree and the identified Yogurt. Finally, participants who always look for natural ingredients in snack foods perceived the products to be more natural, though there was no significant difference for the unidentified Puree, unidentified Cheetos, and the identified Yogurt.

### 3.3. Australia

The fruit-based products were perceived to be the most natural and there were no significant differences between the identified and unidentified (Figure 1). The identified Blueberry Yogurt (Natural Color, Artificial Flavor) was perceived as natural by 47% of Australian respondents and the identified Strawberry Puree (Natural Color, Natural Flavor) was perceived as natural by 43% of respondents (Figure 1). When unidentified, both the Yogurt and Puree were perceived to be natural by 41% of respondents. There was no significant difference between the unidentified Flamin’ Hot Cheetos^®^ (Artificial Color, Natural Flavor) and the unidentified Gummy Candy (Artificial Color, Artificial Flavor), perceived natural by 25 and 19% of respondents, respectively. There was also no significant difference between the unidentified Gummy Candy and the identified Cheetos (20% natural) and the identified Gummy Candy (19% natural).

Males perceived the products to be more natural and females perceived the products to be less natural, but the difference was not significant for the unidentified Puree and the identified Yogurt. Millennials and Centennials perceived products to be more natural than respondents in the other generational groups. These differences were not significant for the identified and unidentified Puree and the identified Yogurt. There were only significant Race/Ethnicity differences for the identified and unidentified Cheetos and the identified and unidentified Gummy Candy statements. White/Caucasian respondents perceived these products to be less natural than other groups in this demographic. Respondents with more education perceived the products to be more natural, though there were no significant differences for the identified Puree and Yogurt statements. Income was not a good predictor of naturalness. There were only significant differences for the identified and unidentified Cheetos and the unidentified Gummy Candy and respondents in the middle-income brackets perceived these statements to be more natural. There were no significant differences for any products based on number of children.

There were no significant differences for frequency of reading ingredient statements for any of the products. Similarly, the identified Gummy Candy was the only product with a significant difference for attention to the source of color. Though this product contains artificial color, participants who pay attention to color source most of the time perceived this product to be more natural. Australian respondents who pay attention to the source of flavor more frequently perceived the products to be more natural. There were no significant differences for the identified Puree and the identified and unidentified Yogurt (which contains artificial flavor). There were no significant differences for any of the products based on the importance of the source of color or the source of flavor. Additionally, there were no significant differences for any of the products based on the likelihood not to purchase artificially colored or artificially flavored products.

In general, Australian participants who eat commonly colored foods more frequently perceived the products to be more natural. There were no significant differences for Ice Cream, Gum, Cereal, Yogurt, Fruit Juice, and Soda for the unidentified Puree; Mints, Cereal, Juice, and Soda for the unidentified Cheetos; Cereal, Dressing, Yogurt, and Soda for unidentified Gummy Candy; Hard Candy, Ice Cream, Gum, Cereal, Dressing, Yogurt, Juice, and Soda for unidentified Yogurt; Soda for the identified Puree; Cereal and Soda for the identified Cheetos; Cereal and Yogurt for the identified Gummy Candy; and Hard Candy, Popsicles, Gum, Juice, Soda, and Sports Drinks for the identified Yogurt. Breakfast Cereal, Yogurt, Fruit Juice, and Soda consumption were poor predictors of naturalness ratings for Australian participants. Those that state that they are particular about the healthiness of food perceived the products to be more natural, but there were no significant differences for the identified Puree and identified Gummy Candy. Twenty-one percent of Australian respondents believe that artificially colored and artificially flavored foods are not harmful for health. These people perceived all of the statements to be natural. There were no significant differences for the statement “I try to eat foods that do not contain additives”. Participants who would like to eat only organically grown vegetables perceived the unidentified Puree and unidentified Cheetos to be more natural. Those who look for only Non-GMO ingredients perceived the identified Cheetos to be more natural. Australian respondents who always look for natural ingredients in snacks perceived the unidentified Puree to be more natural than those who do not.

### 3.4. Cross Country Comparisons

Respondents from the US gave the highest mean naturalness scores for all products but the unidentified Strawberry Puree (Table 3). Of the three regions, US respondents gave the lowest mean score to this product, though the difference was not significant. Overall, there was a significant difference by region, with the US scoring significantly higher on average than respondents from the UK and Australia. There was no significant difference between the latter two. Of the products, the identified Puree and Yogurt received higher mean naturalness scores than the unidentified for all three regions. Conversely, the identified Cheetos and Gummy Candy received lower mean naturalness scores than the unidentified. There were two distinct groupings, with the identified and unidentified Puree and Yogurt being significantly different from the identified and unidentified Cheetos and Gummy Candy. The US and Australia gave higher mean scores to the identified Yogurt and the UK gave about the same mean score to the Puree and Yogurt. In the grouping with the lower mean scores, the US and Australia gave higher mean scores to the unidentified Cheetos statement and the UK gave higher mean scores to the unidentified Gummy Candy statement. All three regions gave the lowest mean naturalness scores to the identified Gummy Candy.

When considering demographics, there were not large differences between the US, UK, and Australia. It appears that Males, Millennials, and consumers with more education and higher income are the most likely to give higher naturalness scores that others in their respective demographic groups. This trend was seen in all three regions with the exception of income not being a good predictor of naturalness scores in Australia. Race/Ethnicity was a poor predictor of natural perceptions in all three regions. Additionally, it appears that frequent consumptions of some commonly colored foods and beverages may be good predictors of natural perceptions, but more so for Americans.

Australian respondents made the most selections and Americans made the fewest selections when choosing which sources are appropriate sources of colors and flavors in foods and beverages (Table 4 and Table 5). Only a few sources were selected by more than 50% of the population for each country, however, there were a number of ingredients that were selected as appropriate sources of colors or flavors by 25% or more of the respondents within a country. Respondents from all three regions associated Fruit, Fruit Juice, Vegetables, and Flowers with acceptable sources of food colors and flavors. Generally, ingredients appropriate as food colors also were appropriate as food flavors although some differences were noted. For example, although they were chosen by fewer than 50% of respondents, Algae, Beans, Minerals, Roots, Food Dyes, and Bark were associated with acceptable sources of food colors, but beans were more associated with food flavors and food dyes were more associated with colors. Seaweed was selected by all three regions as an acceptable color or flavor source but was more associated with UK and Australian respondents. Americans strongly associated Extracts with being acceptable color sources. UK respondents were less likely, however, to select Grains. Australian respondents associated Leaves as being an acceptable color source. Chemicals, Meat, Vitamins, Animal Skins or Bones, Clay, and Beneficial Australian respondents also had the most selections for appropriate sources of flavor in foods and beverages and Americans made the fewest selections. All three regions strongly associated Fruit, Fruit Juice, and Vegetables with being appropriate sources of food flavors. They also associated Algae, Meat, Flowers, Beans, Extracts, Minerals, Roots, Grains, Seaweed, and Leaves with being acceptable. Respondents from the UK and Australia selected Flowers and Seaweed more frequently than American respondents. Americans were more associated with Extracts and Vitamins and UK respondents were more associated Insects as acceptable flavor sources. Australians were more associated with Beans, Leaves, Clay, and Beneficial Microorganisms. Insects, Chemicals, Food Dyes, Clay, and Beneficial microorganisms were selected by fewer than 25% of respondents from all three regions, with Beneficial Microorganisms being the lest selected option.

Animal Skins and Bones also were selected by fewer than 25% of Americans, Bark was selected by fewer than 25% of UK respondents, and Vitamins were selected by fewer than 25% of UK and Australian respondents.

## 4. Discussion

Respondents from all three regions perceived the Strawberry Puree (Natural Color, Natural Flavor) and the Blueberry Yogurt (Natural Color, Artificial Flavor) to be more natural than the Flamin’ Hot Cheetos^®^ (Artificial Color, Natural Flavor) and the Gummy Candy (Artificial Color, Artificial Flavor). Both statements, in addition to having plant-based ingredients (Strawberry Puree Concentrate, Blueberries, Fruit and Vegetable Juice), are shorter ingredient statements. Both contain about 15 ingredients, which is much shorter than the Cheetos statement that contains over 20. Of the four statements, the Gummy Candy is the shortest. This not only suggests that products with shorter ingredient statements are perceived as more natural than those with long statements, but also that consumers look at statement length and ingredients within the statement to make decisions about food naturalness. Though the gummy candy statement was the shortest, it contains artificial colors, artificial flavors, and ingredients with chemical sounding names. This combination outweighed the length of the statement and the product was perceived to be less natural. Comparing the naturalness scores of Cheetos and Yogurt, it may be possible that artificial flavors are more acceptable additives than artificial colors. Artificial colors are commonly associated with health conditions like attention deficit disorder and attention deficit hyperactivity disorder. [32], so it may be this negative association that gives greater weight to colorants on perceptions of naturalness. It may also be because artificial colorants are more clearly listed on ingredient statements, whereas flavors are simply listed as “artificial flavors”. A chain of multiple artificial colors (such as Yellow 5, Red 40, Yellow 6, Blue 1, from the Gummy Candy statement) is more visible and therefore more influential.

The Cheetos and Gummy Candy statements were perceived to be significantly less natural by participants from all three regions. Their ingredient statements contain artificial colors, which are clearly stated using their chemical names (Red 40, for example). Including the identity of the statement appears to have an impact on perceptions of naturalness. Adding the product identity increased naturalness scores for the Puree and Yogurt and decreased naturalness scores for the Cheetos and the Gummy Candy. This may indicate that the product as a whole is primarily how consumers make judgements about product naturalness. Secondary to this is the individual parts, or ingredients, that make up the whole. The Strawberry Puree, for example, contains ingredients like Monocalcium Phosphate, Sodium Alginate, and Methylcellulose. Chambers et al. found that consumer perceived ingredients with chemical sounding names to be less natural than ingredients with common names (Sodium Bicarbonate vs. Baking Soda, for example) [33]. Though participants could see these chemical sounding ingredients in the Puree statement, the name Strawberry Puree increased their perceptions of naturalness. When unidentified, respondents from the UK and Australia gave slightly higher scores to the Puree, the statement with Natural Colors and Natural Flavors. Americans, on the other hand, still perceived the yogurt to be more natural than the Puree. This indicates that ingredients in the statement affect perceptions and it is more than just the color and flavor additives that consumers use as clues of product naturalness. This supports Evans’ conclusion that food content is important in perceptions of product naturalness [34]. In addition to containing artificial colors (and artificial flavors in the Gummy Candy), the Cheetos and Candy statements contain chemical sounding ingredients or ingredients that may be novel to consumers. This could explain why these statements, even when unidentified, received significantly lower naturalness scores. When identified, the scores dropped even lower. Chambers also found that novelty of ingredients also affects perceptions of naturalness, as sorghum flour was rated as less natural than wheat flour [33].

The Cheetos and Gummy Candy are also likely associated with more processing. Rozin discovered that highly processed products were perceived as less natural than products with less processing [35]. Strawberry Puree mostly involves physical processing, which was found by Rozin to be more natural to consumers than chemical processing [36]. Blueberry Yogurt is produced though fermentation, which consumer may see as being more natural than extrusion or gel formation. An additional explanation for the decrease in the score when the Cheetos and Gummy Candy were identified could be related to health. Compared to Strawberry Puree and Blueberry Yogurt, Cheetos and Gummy Candy are not healthy products. Dominick found that 63% of their survey participants associated natural foods with “Improved nutritional value” [37]. A snack product and candy product are not commonly associated with being nutritious foods and it may be because of this that they received lower naturalness scores once identified. Forty-eight percent of American respondents, 45% of UK respondents, and 57% of Australian respondents disagreed with the statements “In my opinion, artificially colored/flavored foods are not harmful for my health”. There is still a large group of consumers that is concerned about color and flavor additives in foods and beverages. This likely influenced naturalness scores for the two statements that contain four artificial colors each along with artificial flavors in the Candy. Respondents who agreed with the statement were more likely to perceive all of the products as natural.

Thoughts about appropriate sources of natural colors and flavors were similar between the US, UK, and Australia. Of the three countries, Australians made more selections for both color and flavor sources and Americans made the least. This could mean that Americans are more particular about colorants and flavorants or that Australians are more open minded about natural additive sources. It could also mean that Americans are less willing to participate in Check All That Apply questions. Respondents believe that plant derived additives are much more appropriate than animal, insect, or microbial derived additives or additives than come from the earth, like minerals. The most selected color and flavor sources include Fruit, Fruit Juice, Vegetables, and Flowers. These results mostly align with the FDA’s definition of natural flavor [1]. The largest discrepancy was that participants from the UK associated insects as being an appropriate source of color much more frequently than Americans and Australians. This could indicate that respondents from the UK are less prone to neophobia and are more accepting of the use of insects as ingredients in foods and beverages. It must be noted that the categories were broad, and no examples were given. Thus, for items like bark, where fewer than 30% of consumers in any country indicated they were an appropriate source for either color or flavor, the scores are based only on their interpretation of what bark is. Bark, as a source is not noted as acceptable, but an example such as cinnamon might be viewed as much more appropriate. Other studies [7,33] have shown consumer confusion about ingredients and their sources impacts the perception of natural.

There were some limitations with this study. Only four ingredient statements were used to measure perceptions of naturalness. In addition, all of the products used were food products. More research could be done using more products and beverages to verify the results of the present study. Only three English-speaking countries participated in the online survey. Further research is needed to understand the naturalness perceptions of consumers from Latin America, Africa, Asia, and European countries other than the UK.

## 5. Conclusions

The results from this experiment illustrate that there are many cues that consumers use when determining the naturalness of a food or beverage. Possibly the most important factor is the product as a whole. When statements were identified as actual products, naturalness ratings for the Strawberry Puree and Blueberry yogurt increase whereas naturalness ratings for the Flamin’ Hot Cheetos^®^ and Gummy Candy decreased. The presence of artificial colors and artificial flavors appears to have an impact on naturalness perceptions, but other ingredients or additives also influence perceptions. Between artificial colors and flavors, the former may influence naturalness perceptions more so than flavors. This could possibly be due to negative health associations with artificial colors or the manner in which they are listed on ingredient statements. In addition to colorants and flavorants affecting perceptions of naturalness, ingredients with chemical sounding names and novel ingredients also influence consumers. Along with ingredient content, manufacturing process also has an effect. The two products that underwent processes that are probably less readily known by consumers, Cheetos and Gummy Candy, were perceived to be less natural than the products like puree or yogurt where consumers may have an idea of the processing method.. Finally, the perceived healthiness of a product likely impacts consumer beliefs about naturalness as the less healthful products were deemed less natural than the fruit-based products. All of these factors combine to form the idea of naturalness in the mind of a consumer.

For scientists, manufacturers, and governments, this information helps in understanding the conceptual basis for consumer beliefs related to perceptions of natural. Informational programming, labeling, and other aspects of consumer education may be adapted based on the results of this study. For example, more use of ingredient names understood by consumers on the label or better use of the source or purpose of the ingredient on the label could help consumers better understand the ingredient and whether they consider it natural.

## Figures and Tables

**Table 1:** Demographic percentages *.

Demographic Characteristics	Categories	US	UK	Australia
Gender	Male	48%	50%	48%
	Female	52%	50%	52%
Age	Centennials/Gen Z	25%	15%	14%
	Millennials/Gen Y	18%	23%	24%
	Gen X	26%	26%	26%
	Baby Boomers	20%	26%	26%
	Silent Generation	11%	10%	10%
Race/Ethnicity	American Indian/Alaska Native	1%		
	Asian	4%	5%	12%
	Black/African/Caribbean	10%	3%	1%
	Hispanic/Latino	6%	0%	1%
	Caucasian/White	77%	90%	82%
	Aboriginal/Torres Strait			1%
	Pacific Islander	0%		1%
	Other	1%	<1%	1%
	Prefer Not to Answer	1%	2%	1%
Education	High School or Less	25%	34%	33%
	Associate Degree/Some College	32%	18%	25%
	College Degree	27%	33%	27%
	Post-Graduate	16%	13%	15%
	Prefer Not to Answer	1%	2%	1%
Number of Children currently living in household	No Children	66%	64%	63%
	One Child	14%	16%	16%
	Two Children	16%	14%	15%
	Three Children	3%	5%	4%
	Four or More Children	2%	1%	2%

* Percentages were based on 932, 959, and 969 respondents from the US, UK, and AUS, respectively.

**Table 2:** Income demographic percentages *.

Demographic Characteristics	Categories	US	UK	Australia
Income	Less than $52,000	27%		
	$52–103,999	25%		
	$104–155,999	17%		
	$156–207,999	12%		
	$208–259,999	10%		
	$260,000 or more	6%		
	Less than £20,000		33%	
	£20,000–39,999		33%	
	£40,000–59,999		16%	
	£60,000–79,999		5%	
	£80,000–99,999		4%	
	£100,000 or more		3%	
	Less than $52,000			39%
	$52–103,999			32%
	$104–155,999			15%
	$156–207,999			5%
	$208–259,999			1%
	$260,000 or more			1%
	Prefer Not to Answer	3%	6%	7%

* Percentages were based on 932, 959, and 969 respondents from the US, UK, and AUS, respectively.

**Table 3:** ANOVA results of US, UK, and Australian participants’ naturalness scores to identified and unidentified products with various combinations of artificial (art) and natural (nat) colors and flavors.

Statement/Label	Country		
	USA *	UK *	Australia *
Blueberry Yogurt (Nat color, Art flavor)	5.55 a	5.03 bcde	5.21 abc
Blind Yogurt (Nat color, Art flavor)	5.32 ab	4.75 e	4.94 cde
Strawberry Puree (Nat color, Nat flavor)	5.19 bcd	5.04 bcde	5.06 bcde
Blind Puree (Nat color, Nat flavor)	4.84 de	4.92 cde	4.97 bcde
Flamin’ Hot Cheetos (Art color, Nat flavor)	3.98 fgh	3.74 ghi	3.68 hi
Blind Cheetos (Art color, Nat flavor)	4.31 f	3.94 gh	3.98 fgh
Gummy Candy (Art color, Art flavor)	3.91 gh	3.65 hi	3.53 i
Blind Candy (Art color, Art flavor)	4.06 fg	3.98 fgh	3.86 ghi

* Means with a common letter are not significantly different (regardless of row or column) *p* ≤ 0.05.

**Table 4:** Percentages of US, UK, and AUS respondents rating various color sources as acceptable for natural foods and beverages.

Color Sources	US	UK	Australia
Fruit Juice	64%	63%	66%
Fruit	76%	78%	80%
Insects	17%	35%	26%
Chemicals	20%	22%	23%
Algae	33%	34%	35%
Vegetables	70%	75%	79%
Meat	23%	22%	25%
Flowers	52%	60%	64%
Beans	38%	35%	38%
Extracts	52%	49%	50%
Vitamins	23%	17%	19%
Minerals	30%	29%	32%
Animal Skins or Bones	12%	13%	15%
Roots	44%	46%	49%
Food Dyes	36%	42%	45%
Grains	26%	21%	27%
Clay	14%	16%	18%
Seaweed	41%	52%	56%
Beneficial Microorganisms	10%	11%	11%
Leaves	42%	47%	55%
Bark	26%	26%	28%

**Table 5:** Percentages of US, UK, and AUS respondents rating various flavor sources as acceptable for natural foods and beverages.

Flavor Sources	US	UK	Australia
Fruit Juice	68%	65%	68%
Fruit	80%	79%	81%
Insects	17%	24%	23%
Chemicals	9%	9%	11%
Algae	29%	32%	33%
Vegetables	73%	76%	81%
Meat	42%	42%	49%
Flowers	45%	50%	57%
Beans	49%	49%	54%
Extracts	46%	42%	43%
Vitamins	26%	21%	20%
Minerals	31%	32%	33%
Animal Skins or Bones	21%	25%	27%
Roots	49%	48%	56%
Food Dyes	11%	13%	14%
Grains	46%	39%	46%
Clay	9%	11%	12%
Seaweed	42%	55%	59%
Beneficial Microorganisms	10%	14%	15%
Leaves	41%	45%	54%
Bark	26%	25%	28%
